# Supplementary material for: Arabidopsis Toxicos en Levadura 12 (ATL12): A Gene Involved in Chitin-Induced, Hormone-Related and NADPH Oxidase-Mediated Defense Responses
Source: J Fungi (Basel). 2021 Oct 19;7(10):883. doi: 10.3390/jof7100883 (PMC8540536; doi:10.3390/jof7100883)
Supplement: Supplementary file 1 [file jof-07-00883-s001.zip › Supplementary materials Figure S1.pdf]

```
1 ttatt gcg tct aaa aac atc ttt ctg tcc aaa ttc ata aac acg act ttt ttt gtt ttc gtc

63 ttt ttc tca tcg aat cac cat cct aaa aca atg aat tca cca caa gaa atc tcc att ctc
1                                           M   N   S   P   Q   E   I   S   I   L
123 ttc ttc ttc atc atc ttc tta gac tat gta tct gct caa tct cct cct cct cct aat cta
 11  F   F   F   I   I   F   L   D   Y   V   S   A   Q   S   P   P   P   P   N   L
183 tac gca aca agc gac ctt ttc aag cca agc tta gct atc atc acc gga gtt ttc tcc atc
 31  Y   A   T   S   D   L   F   K   P   S   L   A   I   I   T   G   V   F   S   I
243 gtc ttc act ctc acc ttt gtt ctc ctc gtc tac gcg aaa tgc ttc cac aat gac ctc cga
 51  V   F   T   L   T   F   V   L   L   V   Y   A   K   C   F   H   N   D   L   R
303 tca gag acc gac agt gat gga gaa aga ata cga cat gat cgt ctc tgg caa gga ctt ttc
 71  S   E   T   D   S   D   G   E   R   I   R   H   D   R   L   W   Q   G   L   F
363 aat cgg tct tca aga ttc tct ggt ctt gac aag aaa gca atc gaa tct ctt ccc ttt ttc
 91  N   R   S   S   R   F   S   G   L   D   K   K   A   I   E   S   L   P   F   F
423 aga ttc tct gct ttg aaa gga ttg aaa caa ggg ctt gaa tgt tct gtt tgt ttg tct aaa
111  R   F   S   A   L   K   G   L   K   Q   G   L   E   C   S   V   C   L   S   K
483 ttc gaa gat gtt gag att ctt agg ctt ttg cct aaa tgc aga cac gct ttt cat att ggt
131  F   E   D   V   E   I   L   R   L   L   P   K   C   R   H   A   F   H   I   G
543 tgt att gac cag tgg ctt gag cag cac gct acg tgt cct ttg tgt aga aac aga gtc aat
151  C   I   D   Q   W   L   E   Q   H   A   T   C   P   L   C   R   N   R   V   N
603 att gaa gac gac ctc tct gtg ttg ggt aat agc agc act agt ctt agg atc ttg aac caa
171  I   E   D   D   L   S   V   L   G   N   S   S   T   S   L   R   I   L   N   Q
663 tct gag aca aga gaa gaa gac tcg agg ttg gag att tac att gaa cgc gaa gaa gga act
191  S   E   T   R   E   E   D   S   R   L   E   I   Y   I   E   R   E   E   G   T
723 aat cga ggg tca tca aga ttc agc agt ttc cga aag att cta aag aaa tcg ttg ttg ctt
211  N   D   G   S   S   R   F   S   S   F   R   K   I   L   K   K   S   L   L   L
783 gag cga gaa gga aat gag aac atc gat gag aag aaa ctt atg cac aag ttc aat cac agg
231  E   R   E   G   N   E   N   I   D   E   K   K   L   M   H   K   F   N   H   R
843 atc gtt gtt tcg gat gct gtg ttt aag aac cgc tgg agt aat atc act tca tct gat ttg
251  I   V   V   S   D   A   V   F   K   N   R   W   S   N   I   T   S   S   D   L
903 acg ttt ttg aca tca gaa atg ctg aat tcg gtg tca agc gac aga ttt tcg tcg gtg gat
271  T   F   L   T   S   E   M   L   N   S   V   S   S   D   R   F   S   S   V   D
963 cga gtg cat aga ggt aac ttg cgg gac aag gaa gat atg gaa atg aaa aga atg ttg att
291  R   V   H   R   G   N   L   R   D   K   E   D   M   E   M   K   R   M   L   I
1023 aag cac aaa gac tca tcc aga aga acg gtt tct gag atc acg act gtt tca aga gaa aag
 311  K   H   K   D   S   S   R   R   T   V   S   E   I   T   T   V   S   R   E   K
1083 gcg gtt gga gga agc tac aga ggt tca acc gcg tcc act tcg cag aat tat gcc gtt act
 331  A   V   G   G   S   Y   R   G   S   T   A   S   T   S   Q   N   Y   A   V   T
1143 gcg acc acg gaa gaa aga aga cgg cgg tta tgg ctg ccg atc gct aga aga acg gct cag
 351  A   T   T   E   E   R   R   R   R   L   W   L   P   I   A   R   R   T   A   Q
1203 tgg ttt gtt aac aga gag aaa agt aat gac cta aac acg aca cgc caa aat ctt aat gta
 371  W   F   V   N   R   E   K   S   N   D   L   N   T   T   R   Q   N   L   N   V
1263 tag ttt ttt ccc ctt taa cac aat tta caa aaa aaa atc tca tag tgc att tta aga gtt
 -

1323 tgt tga ata act aaa act aga atg gtc ctg gct ttt agg gtt gac ttt atg tat aaa ctc

1383 gtt atg gtt ccg gat ttg caa ata ctc ttt ggg gac cat ttg atc ggt cac ttg ggt ttt

1443 ggc caa cat gtt tta cat gtg aaa ttt gtt gta gta gac ttg caa gaa aac tct tta cta

1503 ata
```

**Supplement Figure S1: The complete cDNA and amino acid sequences of the *ATL12*.** The solid boxes indicate the start codon and the dashed line box indicates the stop codon. The solid underlined sequences are transmembrane domain 1 and the dashed line is transmembrane domain 2. The thicker solid underlined sequence is the RING domain.
