# Supplementary material for: Arabidopsis Toxicos en Levadura 12 (ATL12): A Gene Involved in Chitin-Induced, Hormone-Related and NADPH Oxidase-Mediated Defense Responses
Source: J Fungi (Basel). 2021 Oct 19;7(10):883. doi: 10.3390/jof7100883 (PMC8540536; doi:10.3390/jof7100883)
Supplement: Supplementary file 1 [file jof-07-00883-s001.zip › Supplementary materials Figure S2.pdf]

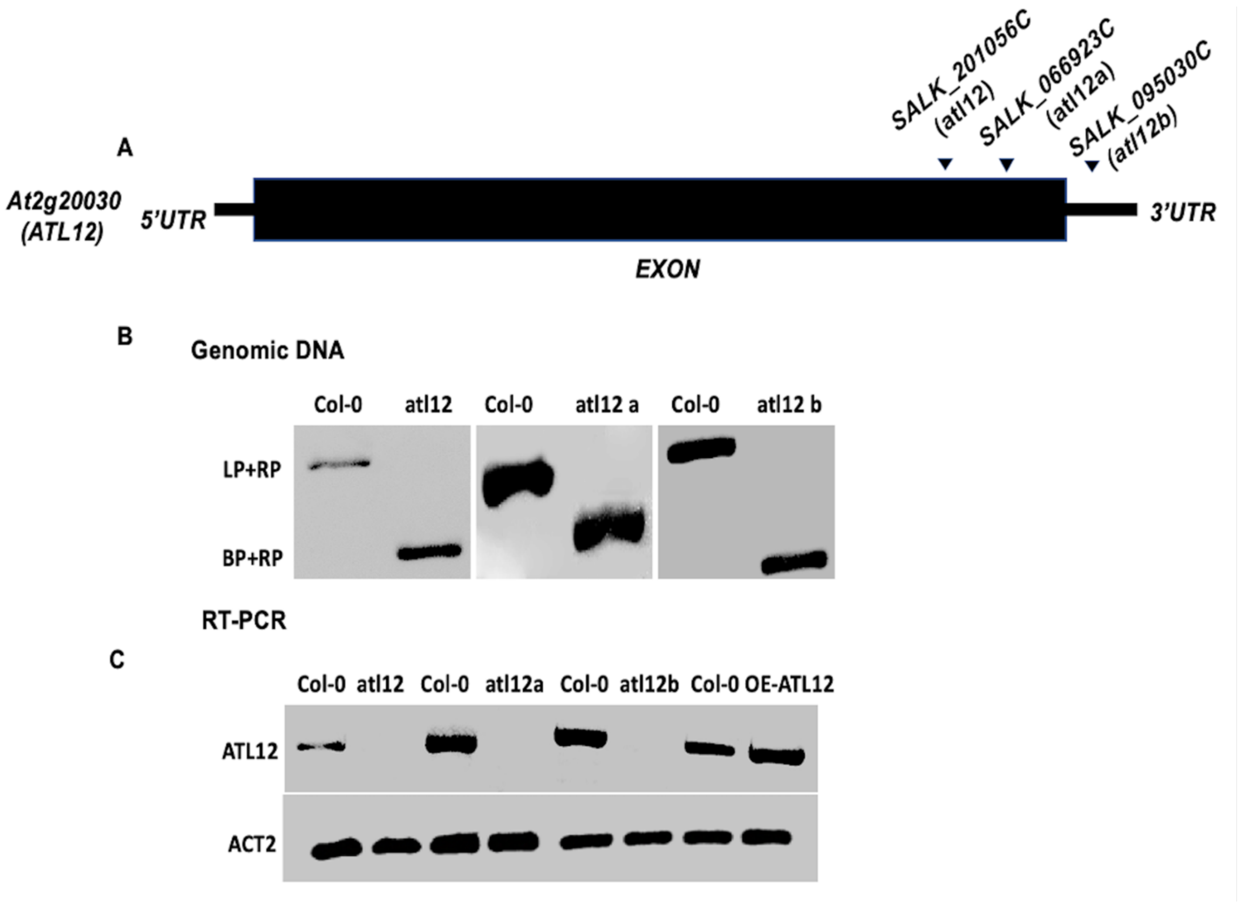

Figure S2. Screen for homozygous T-DNA insertion mutants and overexpression lines.
A. Analyses of T-DNA mutants in the AT2G20030 gene. The locations of the T-DNA insertions in the AT2G20030 gene, SALK_066923C, SALK_095030C, and SALK_201056C, are shown by arrowheads. **B.** Amplification of ATL12 in genomic DNA from the wild type, atl12, atl12a, and atl12b. LP and RP are mutant-specific gene primers. BP is border primer in T-DNA insertional chunk in mutant. **C.** RT-PCR analysis of ATL12 expression in the wild type, the mutants, and overexpression seedlings.
