# Supplementary material for: Arabidopsis Toxicos en Levadura 12 (ATL12): A Gene Involved in Chitin-Induced, Hormone-Related and NADPH Oxidase-Mediated Defense Responses
Source: J Fungi (Basel). 2021 Oct 19;7(10):883. doi: 10.3390/jof7100883 (PMC8540536; doi:10.3390/jof7100883)
Supplement: Supplementary file 1 [file jof-07-00883-s001.zip › Supplementary materials Figure S3.pdf]

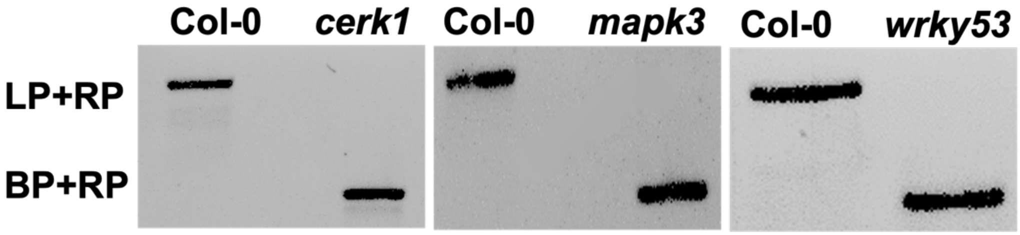

Figure S3. Screening for homozygous cerk1,mapk3 and wrky53 mutants. Amplification of *ATL12* in genomic DNA from the wild type, mutants of *cerk1, mapk3* , and *wrky53*. LP and RP are mutant-specific gene primers. BP is the border primer in T-DNA insertional chunk in the mutant.
